# Fast versus slow weight loss: development process and rationale behind the dietary interventions for the TEMPO Diet Trial

**DOI:** 10.1002/osp4.48

**Published:** 2016-05-31

**Authors:** A. A. Gibson, R. V. Seimon, J. Franklin, T. P. Markovic, N. M. Byrne, E. Manson, I. D. Caterson, A. Sainsbury

**Affiliations:** ^1^ Boden Institute of Obesity, Nutrition, Exercise & Eating Disorders, Sydney Medical School, Charles Perkins Centre University of Sydney Sydney NSW Australia; ^2^ Metabolism & Obesity Services Royal Prince Alfred Hospital Camperdown NSW Australia; ^3^ Bond Institute of Health and Sport, Faculty of Health Sciences and Medicine Bond University Gold Coast Australia

**Keywords:** Clinical protocol, dietary protein, diet‐reducing, energy intake

## Abstract

**Objective and methods:**

Finding effective solutions to curb the obesity epidemic is a great global public health challenge. The need for long‐term follow‐up necessitates weight loss trials conducted in real‐world settings, outside the confines of tightly controlled laboratory or clinic conditions. Given the complexity of eating behaviour and the food supply, this makes the process of designing a practical dietary intervention that stands up to scientific rigor difficult. Detailed information about the dietary intervention itself, as well as the *process* of developing the final intervention and its underlying rationale, is rarely reported in scientific weight management publications but is valuable and essential for translating research into practice. Thus, this paper describes the design *process* and underlying rationale behind the dietary interventions in an exemplar weight loss trial – the TEMPO Diet Trial (Type of Energy Manipulation for Promoting optimal metabolic health and body composition in Obesity). This trial assesses the long‐term effects of fast versus slow weight loss on adiposity, fat free mass, muscle strength and bone density in women with obesity (body mass index 30–40 kg m^−2^) that are 45–65 years of age, postmenopausal and sedentary.

**Results and conclusions:**

This paper is intended as a resource for researchers and/or clinicians to illustrate how theoretical values based on a hypothesis can be translated into a dietary weight loss intervention to be used in free‐living women of varying sizes.

## Introduction

Seeking effective solutions to treat the obesity epidemic is one of the greatest health challenges facing countries all over the world. Increasingly, obesity is regarded as a disease in its own right 1, 2, 3 and is also a risk factor for a large number of non‐communicable, metabolic and mechanical disorders, the risk of which increases on a continuum with increasing adiposity 4. Lifestyle modification – dietary interventions, alterations in physical activity and behavioural changes – remains the cornerstone of obesity management especially in the community 5. Consequently, dietary weight loss trials are an important area of research for informing evidence‐based obesity treatments in clinical practice.

The process of translating evidence from research into clinical practice can take many years 6. An important distinction to make when looking at evidence to inform clinical practice is whether a study is an efficacy or an effectiveness study. Although difficult to completely differentiate between the two, efficacy studies are usually conducted within the confines of tightly controlled laboratory conditions and in a narrowly defined population, with a focus on maximizing internal validity by minimizing confounding variables 7. Effectiveness studies, on the other hand, are conducted in ‘real‐world’ settings and with a broader population, with a focus on maximizing external validity or generalizability to the population at large 7. Effectiveness studies are important for weight management interventions to allow greater generalization of the findings to a primary care setting. An important example of this is the POWER trials (Practice‐based Opportunities for Weight Reduction), a collaboration of three separate primary care interventions with a common focus on weight management and outcome measures 8, 9, 10, 11. These studies have provided evidence that effective weight management interventions can be delivered in a primary care setting. In order to bridge the gap in translation of research into clinical practice, the design of research at all stages (including efficacy studies) should consider the clinical utility of the dietary intervention that is being tested and report greater detail of intervention characteristics 6, 12. If efficacy studies are designed from a practice perspective, this would allow greater translation into effectiveness studies and ultimately into clinical practice 12, 13.

The need for long‐term follow‐up in weight loss trials necessitates that even efficacy trials are conducted in free‐living conditions, outside the confines of tightly controlled laboratory conditions. However, given the complexity of eating behaviour and the food supply, designing a dietary intervention that can be adapted to free‐living conditions, which also stands up to scientific rigor, is difficult. However, such detailed information about the process of designing dietary interventions and their underlying rationale is rarely reported in articles about clinical weight loss trials. Therefore, the aim of this paper describes the design *process* and underlying rationale behind the dietary interventions of an exemplar weight loss trial (details in the succeeding texts). The intention is for this paper to be a resource for researchers and/or clinicians by illustrating how theoretical values based on a hypothesis can be translated into a dietary weight loss intervention to be tested in free‐living women of varying sizes.

## The TEMPO Diet Trial (Type of Energy Manipulation for Promoting optimal metabolic health and body composition in Obesity)

The TEMPO Diet Trial is designed to assess the long‐term effects of fast versus slow weight loss on adiposity, fat free mass, muscle strength and bone density in women who are 45–65 years of age, postmenopausal, sedentary (defined here as less than 3 h of structured physical activity per week) and with obesity (body mass index [BMI] 30–40 kg m^−2^). The trial is registered with the Australia and New Zealand Clinical Trials Registry (ACTRN12612000651886). It is a 3‐year trial consisting of a 1‐year intervention (weight loss) phase, with follow‐up at 2 and 3 years.

The TEMPO Diet Trial is not strictly an efficacy study or an effectiveness study; however, it is conducted in a research setting and in a narrowly defined population and has been designed to maximize clinical utility by drawing on existing resources and clinical practice guidelines (as described in the succeeding texts).

## Modelling of potentially eligible participants

Nutritional requirements differ for persons of different age, sex, height, weight and physical activity level. Hence, the first consideration in designing the dietary interventions for the TEMPO Diet Trial was to model (or predict) characteristics of the potentially eligible population so that accurate dietary prescriptions and the logistics of implementation could be determined prior to seeing participants. A detailed overview of how this was conducted is provided in the Supporting information. In brief, we used the average height of women in Australia in our age group (45–65 years) and in our BMI inclusion criteria (30–40 kg m^−2^) to determine that the weight of potentially eligible women would likely fall between 68 and 115 kg. We then calculated the estimated energy expenditure (EEE) for all potentially eligible women within these weight ranges using the Harris–Benedict equation 14.

## Developing the foundations for the dietary weight loss interventions

### Energy restriction

As the TEMPO Diet Trial compares fast versus slow weight loss, we aimed to achieve a large difference in *energy restriction* between the two intervention arms. We thus established *energy restriction targets* as this would take into account differences between participants in baseline requirements, important for achieving our goal of comparing fast versus slow weight loss. This strategy is in contrast to other common approaches in clinical weight loss trials, which focus on a specific *energy intake target* (e.g. 5000–6300 kJ [1200–1500 kcal] per day) 15. However, if we used an energy intake target, this could result in wide variability in the rate of weight loss within each arm and less differences between arms, because the rate of weight loss is dependent not only on energy intake but also on energy requirements, which can vary widely in a potential participant population (refer to Supporting information, Figure S2, for examples).

We aimed to achieve severe energy restriction and moderate energy restriction in the fast and slow weight loss groups, respectively. We defined severe energy restriction as a minimum of 65% restriction relative to EEE (target range 65–75%) and moderate energy restriction as a maximum of 35% restriction relative to EEE (target range 25–35%). As such, we were aiming for a minimum *difference* in energy restriction between women in the fast versus slow weight loss arms of 30% of EEE.

### Protein intake

An additional requirement in designing the dietary interventions for the TEMPO Diet Trial was to match protein intake between the groups as closely as possible. This is because protein intake has been shown to have a dose–response effect on the composition of weight that is lost during an energy‐restricted diet, as well as appetite 16, 17, 18 – both of which are outcomes of the TEMPO Diet Trial. Further, protein intake needed to be matched on an absolute (gram per kilogram of body weight per day) as opposed to a relative basis (% of energy from protein). This is because of the large differences in prescribed energy restriction (and therefore prescribed energy intake) among potential participants, which, if protein intake were based on a percentage of energy, would result in large absolute differences in protein intake between the fast and slow weight loss groups.

In choosing the absolute protein target to use for the trial, consideration was given not only to the recommended dietary intake (RDI) of protein and the available evidence on protein intakes shown to minimize loss of fat free mass and to attenuate the drive to eat during weight loss but also to what would be feasible within both the fast and slow weight loss arms. The RDI of protein in Australia for women aged 51–70 years is 0.75 g per kg of actual body weight per day 19. In order to prevent or attenuate loss of fat free mass during energy restriction and weight loss in people with overweight or obesity, a protein intake of between 0.8 and 1.2 g per kg of actual body weight per day is suggested 16, 17, 18, 20. The upper level of 1.2 g per kg per day appears to be the most beneficial in terms of body composition 20; however, this was deemed too high for the purposes of this study, as it would not be feasible to achieve this level of protein intake within the fast weight loss arm, given the severe energy restriction involved. Similarly, the minimum of 0.8 g per kg per day was deemed too low for this trial because it is likely that participants in the slow weight loss arm would inadvertently consume more than 0.8 g of protein per kg of body weight per day because of the difficulty of maintaining such a low protein intake with only moderate energy restriction. For instance, the latest Australian National Nutrition Survey found that women aged 51–70 years consumed, on average, 18.9% of energy from protein 21. A potential participant in the trial of average height (1.62 m) and with a BMI of 35 kg m^−2^ would weigh 91 kg and have an EEE of 9900 kJ (Supporting information, Figure S2). Because 1 g of protein provides 17 kJ, 18.9% of energy as protein in weight maintenance would equate to 110 g per day or 1.2 g per kg body weight. Therefore, a target protein intake of 1 g per kg was chosen as it represented a suitable compromise between adequate (0.8 g per kg) and optimal (1.2 g per kg) protein intake that was also *feasible* for both intervention arms.

## How the two dietary weight loss interventions were developed

### Development of the fast weight loss intervention

We chose to achieve fast weight loss via severe energy restriction using commercial formulated meal replacement very low energy diet (VLED) products (as opposed to food). This is because these products would not compromise micronutrient intake and are routinely used in clinical practice. Given our target protein intake of 1 g per kg of actual body weight per day and given the varying body weights of potential participants in the trial, a number of different fast weight loss regimens were needed for the trial. As well as aiming to achieve this target for protein intake, we also needed to ensure that the diet involved severe energy restriction (65–75% of EEE) and that carbohydrate intake was low enough to induce ketosis. Ketosis is a metabolic state involving an elevation in circulating concentrations of ketone bodies and is thought to be a key factor in helping to prevent a compensatory increase in the drive to eat with severely restricted energy intake 22. Whilst the exact level of carbohydrate intake at which ketosis occurs is not known, ketosis is thought to be unlikely when carbohydrate intake is above 100 g per day 23, 24. Thus, in addition to the previously mentioned targets for energy restriction and protein intake, we also wanted to ensure a carbohydrate intake of less than 100 g per day.

When we examined the VLED products currently available in Australia, it became apparent that it was not going to be feasible to achieve a protein intake of 1 g per kg using the products alone without pushing energy and carbohydrate intake above target levels for our trial. For example, a woman with a BMI of 35 kg m^−2^ and of average height for women in Australia of our age group (1.62 m) 25 would weigh 91 kg and require six Optifast® shakes per day (17.5 g protein per sachet, Table 1), resulting in a daily energy intake of 5200 kJ (870 kJ per sachet, Table 1) – which is only 47% energy restriction given her EEE of 9900 kJ per day (Supporting information, Figure S2) – and a daily carbohydrate intake of well over 100 g (135 g, 22.5 g per sachet, Table 1). Therefore, protein supplementation would be required in our fast weight loss intervention. This could potentially be achieved by including a small portion of lean protein in the diet (e.g. beef, chicken or fish). However, the practice of supplementing VLED products with lean protein may decrease compliance 26, 27, as paradoxically, restricting choice to solely the VLED products is what is thought to promote adherence 28. Thus, we used a carbohydrate‐free protein supplement product that could be added to the VLED products. To this end, we selected a whey protein isolate product (Beneprotein®, Nestlé HealthCare Nutrition, Inc., Florham Park, NJ, USA), which readily dissolves in water because it is intended for use in tube feeds, contains no carbohydrate and is unflavoured. This was in contrast to many of the protein supplement products that are readily available in Australian pharmacies, which can contain significant quantities of carbohydrate and are usually flavoured.

**Table 1:** Nutritional composition and cost of the very low energy diet and protein supplement products used to model potential fast weight loss regimes

Product	Energy (kJ)	Protein (g)	Fat (g)	Carbohydrate (g)	Fibre (g)	Cost (AUD)*
Average of one Optifast® sachet (54 g)	870	17.5	4.5	22.5	3.6	3.50
Average of one KicStart® sachet (55 g)	856	22.3	3.8	18.4	3.05	2.95
One scoop of Beneprotein® whey protein isolate (7 g)	105	6	0.0	0.0	0.0	0.62

AUD, Australian Dollars.

* Based on price to the consumer of the only available pack sizes: AUD41.95 for a box of 12 Optifast® shakes; AUD2.95 for a single KicStart™ shake; AUD19.95 for a 270 g can of Beneprotein®.

To determine the most suitable VLED product to use in our trial, we modelled the average nutritional composition of two commercially available products that are readily available, contain sound micronutrient composition and are routinely 29 used as VLEDs (Optifast®, Nestlé Healthcare Nutrition, Rhodes, NSW, Australia; and KicStart™, Prima Health Solutions Pty Ltd., Frenchs Forest, NSW, Australia, Table 1). Nutritional composition and cost in Australian Dollars were taken from the respective manufacturers' websites 30, 31. We chose to only consider the shake varieties of the VLED products and did not include soup, dessert or bar varieties, because in our clinical experience, shakes are the most popular. The average energy and macronutrient content of the Optifast® and KicStart™ shakes and Beneprotein®, as well as price, are shown in Table 1.

To design the different fast weight loss regimens, we generated a series of regimens for each of the VLED products based on varying the number of shakes and scoops of Beneprotein®. We worked backwards from our three main desired features of the diet: a daily protein intake of 68–115 g per day (corresponding to the potential weights of our eligible participants of 68–115 kg), a severe energy restriction of 65–75% of EEE, and a daily carbohydrate intake of less than 100 g. We started with a regimen containing a minimum of three shakes (as three are required to meet micronutrient requirements), and thereafter, each regimen increased by one scoop of Beneprotein® per day (Table 2). We increased Beneprotein® up to a maximum of two scoops per shake (i.e. six per day for a regimen based on three shakes) before devising a regimen with an increased number of shakes per day. The maximum of two scoops of Beneprotein® per shake was based on taste testing in which we found that two scoops could be added during the preparation of a shake before it started to affect the taste. Note that there were no regimens with Optifast® involving less than two scoops of Beneprotein®, as these were required to meet the minimum protein intake of 68 g with Optifast® shakes (Table 2). Once we had regimens that represented all the required protein intake targets of 68–115 g per day, unnecessary regimens were deleted. For example, if a regimen with four shakes resulted in a protein intake that was also met by a regimen with three shakes, we only included the regimen with three shakes, in order to minimize energy and carbohydrate intake, as well as cost. Indeed, the first regimen in Table 2 to include four Optifast® shakes has four scoops of Beneprotein® rather than 0, 1, 2 or 3, as the protein intake of these regimens could be met with regimens including three Optifast® shakes.

**Table 2:** Average daily nutritional composition and cost of two sets of potential fast weight loss regimes modelled using different very low energy diet products to meet protein requirements of 68–115 g per kg of actual body weight per day

Regime no.	No. of shakes	No. of Beneprotein® scoops (7 g)	Energy (kJ)	Protein (g)	Fat (g)	Carbohydrate (g)	Fibre (g)	Total cost (AUD per day)	Total cost (AUD per week)
OPTIFAST® (54 g)									
1	3	2	2820	64.5	13.5	67.5	10.8	11.74	82.18
2	3	3	2925	70.5	13.5	67.5	10.8	12.36	86.52
3	3	4	3030	76.5	13.5	67.5	10.8	12.98	90.86
4	3	5	3135	82.5	13.5	67.5	10.8	13.60	95.20
5	3	6	3240	88.5	13.5	67.5	10.8	14.22	99.54
6	4	4	3900	94.0	18.0	90.0	14.4	16.48	115.36
7	4	5	4005	100.0	18.0	90.0	14.4	17.10	119.70
8	4	6	4110	106.0	18.0	90.0	14.4	17.72	124.04
9	4	7	4215	112.0	18.0	90.0	14.4	18.34	128.38
10	4	8	4320	118	22.5	90.0	14.4	18.96	132.72
KicStart™ (55 g)									
1	3	0	2568	67.0	11.5	55.2	9.2	8.85	61.95
2	3	1	2673	73.0	11.5	55.2	9.2	9.47	66.29
3	3	2	2778	79.0	11.5	55.2	9.2	10.09	70.63
4	3	3	2883	85.0	11.5	55.2	9.2	10.71	74.97
5	3	4	2988	91.0	11.5	55.2	9.2	11.33	79.31
6	3	5	3093	97.0	11.5	55.2	9.2	11.95	83.65
7	3	6	3198	103.0	11.5	55.2	9.2	12.57	87.99
8	4	3	3739	107.3	15.3	73.5	12.2	13.66	95.62
9	4	4	3844	113.3	15.3	73.5	12.2	14.28	99.96
10	4	5	3949	119.3	15.3	73.5	12.2	14.90	104.30

AUD, Australian Dollars.

**Figure 1:**
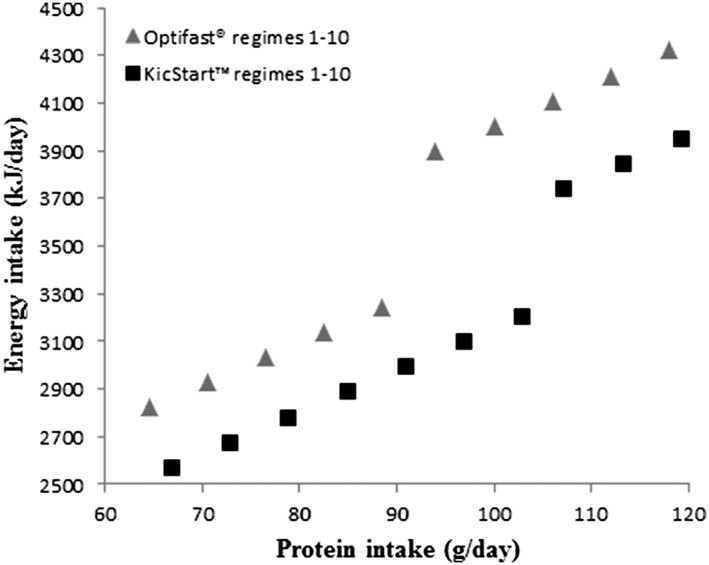
Daily protein intake targets of potential fast weight loss regimes can be met at a lower daily energy intake if KicStart™ very low energy diet products are used as the basis of the regime rather than Optifast® products.

**Figure 2:**
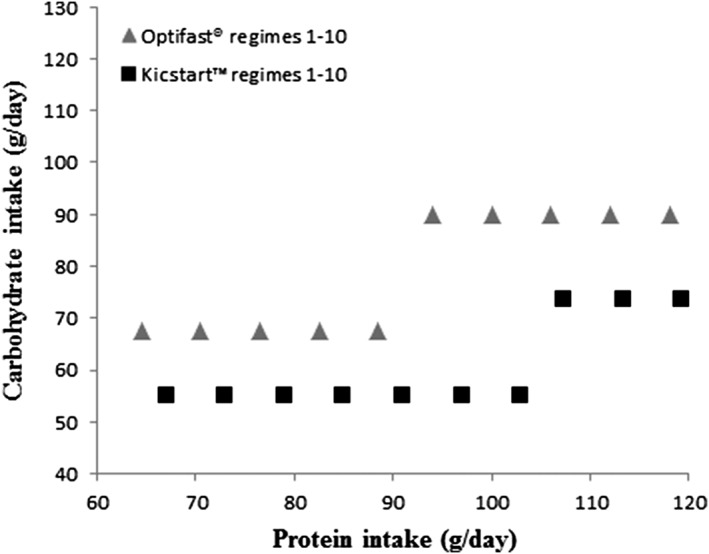
Daily protein intake targets of potential fast weight loss regimes can be met at a lower daily carbohydrate intake if KicStart™ very low energy diet products are used as the basis of the regime rather than Optifast® products.

**Figure 3:**
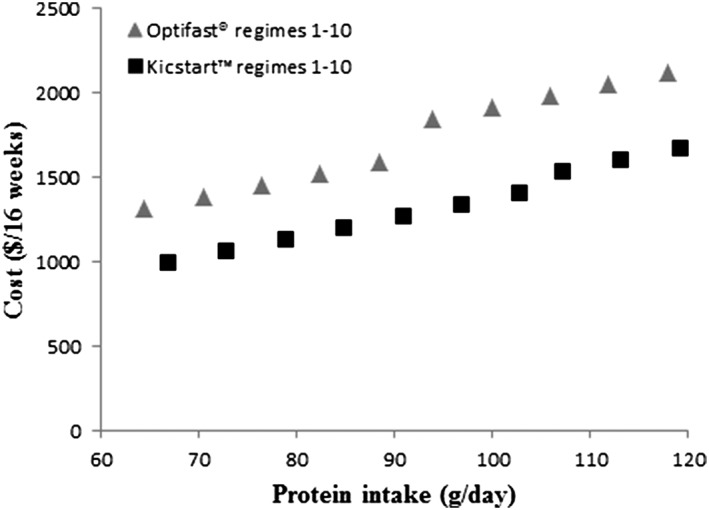
Daily protein intake targets of potential fast weight loss regimes can be met at a lower program cost (16 weeks for this trial) if KicStart™ very low energy diet products are used as the basis of the regime rather than Optifast® products.

To compare the regimens based on the two VLED products shown in Table 2, we plotted the daily energy intake, daily carbohydrate intake and cost for a program of our intervention duration (16 weeks) against the protein intake provided by each regimen (Figures 1, 2, 3). This demonstrated that whilst the two VLED products were comparable in energy content when compared on a per shake basis (Table 1), because the KicStart™ shakes contained an average of 4.8 g more protein, 4.1 g less carbohydrate and retailed at AUD0.55 less than Optifast® shakes (AUD1.17 less if KicStart™ is purchased in bulk), it was better suited to our intervention aims of meeting protein requirements whilst keeping energy, carbohydrate intake and cost within stringent upper limits. For example, if we compare the energy, carbohydrate and cost saving for three women of average height (1.62 m) with a BMI of 30, 35 and 40 kg m^−2^ respectively, using KicStart™ instead of Optifast® results in a daily saving per person of 252–912 kJ, 12.4–34.8 g of carbohydrate and AUD2.89–5.15 (AUD20.23–36.05 per week).

Once we had selected the VLED product to be used in our trial based on the previously mentioned considerations (KicStart™), we recalculated our regimens incorporating vegetable and oil allowances. The vegetable and oil allowance included two cups of non‐starchy vegetables and one teaspoon of fat or oil. Vegetables are recommended in clinical treatment protocols not only to help increase fibre intake, as constipation is a common side effect of VLEDs, but also to help with the social aspects of eating 28. The teaspoon of oil was originally included to cause contraction of the gall bladder and minimize the risk of gall stones 28. Modern formulations of VLED products now contain sufficient fat so the addition of oil to the diet is not essential. However, adding oil to the diet does allow a greater flexibility and variety of dishes to be prepared using the vegetable allowance. Vegetables and oil would not be compulsory for those in the fast weight loss arm in this trial but would be strongly encouraged for the aforementioned reasons. For calculation of the composition of these allowances, we used one third of a cup each of cauliflower, broccoli, zucchini, spinach, green beans and carrots plus one teaspoon of olive oil. The nutritional composition of these foods was 400 kJ, 4.2 g protein, 5.1 g fat, 5.3 g carbohydrate and 6.2 g fibre, which was analysed with FoodWorks® software version 7 (Xyris Software (Australia) Pty Ltd., Kenmore Hills, Australia). The recalculated regimens are shown in Table 3. Note that only regimens 1–8 are shown: Regimens 9 and 10 were no longer required because the protein content of the vegetable allowance made them unnecessary.

**Table 3:** Weight categories, nutritional composition and lowest and highest degree of estimated energy restriction of each of the final fast weight loss regimes using KicStart™ incorporating vegetable and oil allowances

Regime number	Participant weights for which this regime would be used (kg)*	No.of shakes	No. of Beneprotein® scoops (7 g)	Energy (kJ)	Protein (g)	Fat (g)	Carbohydrate (g)	Fibre (g)	Lowest ER† (%)	Highest ER†(%)
1	68–74	3	0	2.97	71	17	60	15	67	69
2	>74–80	3	1	3.07	77	17	60	15	66	69
3	>80–86	3	2	3.18	83	17	60	15	66	69
4	>86–92	3	3	3.28	89	17	60	15	65	69
5	>92–98	3	4	3.39	95	17	60	15	64	67
6	>98–104	3	5	3.49	101	17	60	15	65	67
7	>104–110	3	6	3.60	107	17	60	15	64	66
8	>110–115	4	3	4.14	112	20	79	18	60	61

ER, energy restriction.

* These weight categories were based on participants' heights between 1.5 and 1.7 m tall. As such, potentially eligible participants may fall outside these weight categories, but only if their BMI is on the extremes of our criteria. As such, additional regimes would need to be devised for those heavier than 115 kg.

† To determine the lowest and highest ER provided by each regimen, we used our model of EEE of potential participants (refer to Supporting information, Figure S2) to identify the lowest and highest EEE of the potential participants that would be prescribed that regimen (i.e. the lightest and shortest participants to the heaviest and tallest participants, respectively).

In order to ensure that our fast weight loss regimens were indeed meeting our targets of 65–75% energy restriction, as well as daily protein and carbohydrate intakes of 1.0 g per kg and less than 100 g, respectively, we cross‐checked our eight regimens against our model of potential participants (Table 3). As the regimens differed by 6 g increments of protein (the amount of protein in one 7 g scoop of Beneprotein®), and as the regimen a particular participant would be prescribed would be based on their starting weight, we used the protein content of the regimens to determine weight categories. The upper and lower cut points were taken from the average protein content of two adjacent regimens. As an example, for regimen number 4, protein intake is 89 g per day, and for regimen number 5, protein intake is 95 g per day (Table 3); therefore, the upper cut point of body weight for regimen 4 is less than 92 kg, and the lower cut point of body weight for regimen number 5 is greater than or equal to 92 kg.

Once the weight categories were determined for each regimen, we also cross‐checked the energy intake of each regimen against the range of EEEs of potential participants to be prescribed that regimen, to ensure that it would involve severe energy restriction for those participants. This showed that on average, the energy restriction for each regimen would fall within our required 65–75% energy restriction (Table 3). The exception to this would be the heaviest potential participants (>110), who would require four shakes per day (regimen 8) upon commencement of the intervention, which would result in an energy restriction of 60–61%, which is lower than our target range of 65–75%. In summary, our modelling work has shown that it would be possible to achieve our aims of providing a weight loss diet involving severe energy restriction and less than 100 g carbohydrate per day whilst also meeting our protein target of 1 g per kg of actual body weight per day.

**Table 4:** Average energy and protein provided by a serve of each of the six food groups used to develop the different regimes for the slow weight loss intervention

Food group	Energy (kJ)	Protein (g)
Proteins	550	15
Carbohydrates	500	3
Vegetables	100	2
Fruits	350	1
Fats	350	0
Discretionary	600	2

The fast weight loss intervention in the TEMPO Diet Trial involves 16 weeks of severe energy restriction followed by the same diet as those on the slow weight loss intervention (details in the succeeding texts), for a total of 12 months. As actual body weight would inevitably change over the course of the intervention, the regimens used to achieve fast weight loss would be adjusted every 4 weeks. Participants would receive a booklet with information about the fast weight loss intervention, including a list of allowed extras, answers to frequently asked questions (e.g. side effects and their management) and recipe suggestions for the non‐starchy vegetable allowance.

### Development of the slow weight loss intervention

Whilst there are many different ways to achieve slow weight loss via moderate energy restriction – and the debate about the optimal macronutrient composition of weight loss diets continues 32 – we chose to base our slow weight loss intervention on the Australian Guide to Healthy Eating (AGHE), the Australian Government Dietary Guidelines released in April 2013 33. There are several reasons why the national dietary guidelines of a country are an ideal basis for a weight loss intervention in that country (in this case, Australia), notably because they are based on foods that are typically eaten and easily accessible in that country by a variety of cultural groups, as well as being in line with the general goal of weight loss interventions, as outlined in the succeeding texts.

In addition to targets for energy restriction and protein intake, the long duration of the slow weight loss intervention (12 months) meant that key considerations in designing the regimens to be used for the intervention were flexibility, acceptability to participants and sustainability. The AGHE meets these considerations because of the large amount of choice available within each of the core food groups and combinations thereof, enabling food selections to meet taste, cultural and cost preferences for each participant, including those on vegetarian diets. Moreover, as the guidelines were developed for use by the general public, numerous resources on their interpretation and use have been developed, making them an economical, practical, familiar and convenient tool to use as the basis of our slow weight loss intervention.

As well as the flexibility and convenience of the AGHE, it is also nutritionally sound. The AGHE is a dietary guideline designed to meet nutrient requirements with minimum energy intake – providing the optimum basis for a weight loss intervention. After all, the types of foods that are recommended to people for weight loss (high in nutrients, low in energy) are the same as those that are recommended to the general population for optimal health and well‐being. The latest Australian National Nutrition Survey showed that discretionary foods (not part of the five core food groups) contributed 35–45% of the total energy intake of Australian adults 34. Discretionary foods are typically energy dense, low in protein and high in refined carbohydrates and fat (particularly saturated fat). Further, the latest Australian Health Survey, which was conducted in 2011–2012, found that less than 10% of women aged 45–64 years consumed the recommended two serves of fruit and five serves of vegetables every day 35. Taken together, a decreased consumption of discretionary foods and increased consumption of fruits and vegetables, as recommended by the AGHE, aligns with the requirements of weight loss interventions such as the slow weight loss intervention to be used in the TEMPO Diet Trial.

The AGHE provides recommendations as to the average number of standard serves of the five core food groups an individual should eat in order to meet their nutritional requirements, based on their age and sex. Core foods are those that provide nutrients essential for health, with minimal amounts of added saturated fat, sugar, salt and alcohol. The five core food groups are fruits, vegetables, grains and cereals, meat and meat alternatives and reduced fat dairy. A standard serve is an energy equivalent within a core food group of the AGHE. For example, all of the standard serves of meat and meat alternatives in the AGHE (65 g of cooked red meat, 120 g of firm tofu, etc.) provides between 500 and 600 kJ. For the purposes of our slow weight loss intervention, and to simplify adherence, the meat and meat alternative and reduced fat dairy core food groups of the AGHE were collapsed into a ‘proteins’ group, and starchy vegetables were incorporated into the grains and cereals group to form a ‘carbohydrates’ group. For females, an allowance of 14–20 g per day of spreads and oils based on unsaturated fats is included as part of the AGHE to reflect culinary behaviour. For the purposes of our intervention, we defined a ‘fat’ serve as two teaspoons (10 g) of spread or oil, which provides approximately 350 kJ.

Discretionary choices are defined in the AGHE as foods that are not essential for health and are therefore not part of the core food groups. They include foods that are traditionally referred to as ‘junk foods’, such as biscuits, cakes and ice cream. However, they also include ingredients that may be used in food preparation to add flavour to and increase the palatability of core foods – such as honey added to porridge or a stir‐fry. As such, we considered it important to include one serve (600 kJ) of discretionary foods per day to not only help with compliance for the previously mentioned culinary reasons but also to improve the social aspects of weight loss. For example, participants could ‘save up’ their serves of discretionary foods for a social event. In summary, we used the five core food groups, added fat allowances and discretionary foods from the AGHE to define six food groups for the purposes of this intervention: proteins, carbohydrates, vegetables, fruits, fats and discretionary (Table 4).

To design the regimens for the slow weight loss intervention, we once again worked backwards from the desired output, which was a daily protein intake of 68–115 g per kg of actual body weight per day (corresponding to the potential weights of our participants of 68–115 kg) and an energy restriction of 25–35% of EEE. For this purpose, we used the average energy and protein provided by a serve of each of the six food groups in this intervention, as shown in Table 4. These values were determined as described in the succeeding texts.

Although foods are grouped in Table 4 based on the major types of nutrients they provide, the large number of foods within each of the food groups means that they would inevitably vary in energy and protein content. As such, a number of assumptions were inherent in our model. For the proteins group, which was formed by combining the meat and meat alternatives and reduced fat dairy core food groups of the AGHE, a standard serve for both in the AGHE is 500–600 kJ. As such, we took the midpoint of this range and defined the average energy content of the proteins group as 550 kJ. For the carbohydrates group, which was formed by combining the grains and cereals and starchy vegetables, the energy content of a standard serve in the AGHE is 500 and 350 kJ, respectively. We increased the energy content of a starchy vegetable serve to 500 kJ for consistency. For all other groups, the energy content of a serve was the same as an AGHE serve (Table 4).

To calculate the average protein content of a standard serve of the proteins group, as well as that of the other food groups used for this intervention, we entered a range of standard serves of different foods from that food group into a recipe file in FoodWorks® software version 7 (Xyris Software (Australia) Pty Ltd., Kenmore Hills, Australia) and then divided the total amount of protein by the number of serves. For example, for the proteins group, we entered the following serves into FoodWorks®: 100 g of raw lean beef (22 g of protein, 518 kJ), 100 g of raw lean lamb (22 g of protein, 546 kJ), 100 g of raw skinless chicken breast (22 g of protein, 438 kJ), 100 g of raw lean pork (23 g of protein, 469 kJ), two eggs (13 g of protein, 553 kJ), 100 g of tofu (12 g of protein, 502 kJ), one cup of reduced fat milk (10 g of protein, 528 kJ), 150 g of reduced fat fruit yoghurt (8 g of protein, 551 kJ), one half cup reduced fat ricotta cheese (11 g of protein, 550 kJ) and 40 g of reduced fat cheese (11 g of protein, 561 kJ) and then divided the total amount of protein in all of these 10 serves (154 g) by the number of serves (10) to get an average protein content of 15.4 g per standard serve, which we rounded down to 15 g as shown in Table 4.

To develop the slow weight loss regimens, we used a similar process to that used to develop the fast weight loss regimens, by increasing the amount of protein (in increments of half serves of the proteins group), starting with a minimum of three serves from the proteins group, until we had regimens that met our target range of protein intakes (Table 5). We kept the number of serves of foods from the other groups constant (four serves from the carbohydrates group, five from the vegetables group, two from the fruits group, two from the fats group and one from the discretionary group) – aligning as closely as possible to the number of standard serves per day recommended in the AGHE for a 51–70‐year‐old woman.

**Table 5:** Average predicted protein and energy intake and the lowest and highest predicted energy restriction of each of the slow weight loss regimes

Regime number	Weight of potential participants for which this regime would be used (kg)*	No. of protein serves	No. of carbohydrates/vegetables/fruits/fats/discretionary serves	Energy (kJ)	Protein (g)	Lowest ER† (%)	Highest ER (%)†
1	>68–75	3	4/5/2/2/1	6.4	71	30	33
2	>75–83	3.5	4/5/2/2/1	6.7	79	33	30
3	>83–90	4	4/5/2/2/1	7.0	86	33	28
4	>90–98	4.5	4/5/2/2/1	7.2	94	33	28
5	>98–106	5	4/5/2/2/1	7.5	101	31	27
6	>106–113	5.5	4/5/2/2/1	7.8	109	30	27
7	>113–115	6	4/5/2/2/1	8.1	116	24	24

ER, energy restriction.

* These weight categories were based on participants' heights between 1.5 and 1.7 m tall. As such, potentially eligible participants may fall outside these weight categories, but only if their BMI is on the extremes of our criteria. In which case, additional regimes would need to be devised for those heavier than 115 kg.

† To determine the lowest and highest ER provided by each regimen, we used our model of EEE of potential participants (refer to Supporting information, Figure S2) to identify the lowest and highest EEE of the potential participants that would be prescribed that regimen (i.e. the lightest and shortest participants to the heaviest and tallest participants, respectively).

In order to ensure that our slow weight loss regimens were indeed meeting our target of 25–35% energy restriction and a daily protein intake of 1.0 g per kg, we cross‐checked our seven regimens against our model of potential participants (Table 5). As the regimens differed by 7.5 g increments of protein (the amount of protein in half a serve of foods from the proteins group), and as the regimen a particular participant would be prescribed would be based on their starting weight, we used the protein content of the regimens to determine weight categories. The upper and lower cut points were taken from the average protein content of two adjacent regimens. As an example, for regimen number 3, average protein intake is 86 g per day, and for regimen number 4, average protein intake is 94 g per day (Table 5); therefore, the upper cut point for regimen 3 is a body weight of less than 90 kg, and the lower cut point for regimen 4 is a body weight of greater than or equal to 90 kg.

Once the weight categories were determined for each regimen, we also cross‐checked the energy intake of each regimen against the range of EEEs of potential participants to be prescribed that regimen, to ensure that it would involve moderate energy restriction for those participants. This showed that on average, the energy restriction for each regimen would fall within our required 25–35% energy restriction (Table 5). The exception to this would be the heaviest potential participants (>113–115 kg), who would start on regimen 7 upon commencement of the intervention, which would result in an energy restriction of 24. %, which is lower than our target range of 25–35%. In summary, our modelling work has shown that it would be possible to achieve our aims of providing a weight loss diet involving moderate energy restriction whilst also meeting our protein intake target of 1 g per kg of actual body weight per day.

## Implementation of the fast and slow weight loss interventions

Implementation of an education‐based dietary intervention (as opposed to a dietary intervention where meals are provided) presents multiple challenges. It is well known that people consistently eat similar foods to those they normally eat, even when they are prescribed special diets. For this reason, all participants in the TEMPO Diet Trial would be required to complete a 7‐d food diary prior to beginning the intervention. This would be used not only for data collection of baseline dietary intake but also to individualize the interventions for each participant. Participants would be provided with the Australian Dietary Guideline resources (available from www.eatforhealth.com), as well as individualized meal plans and behavioural resources as appropriate to the intervention to which they are randomized and the stage of the intervention.

Following randomization, all participants would receive oral and written information about the dietary intervention to which they have been randomized. This information would be provided by an Accredited Practicing Dietitian (Australian equivalent of a Registered Dietitian in the UK and USA). The number and timing of individual dietary appointments used in the TEMPO Diet Trial was based on the National Health and Medical Research Council (NHMRC) clinical practice guidelines for ‘active weight management’ of adults with a BMI in the overweight and obese range 36. The recommendations are for appointments every 2 weeks for the first 3 months and for continued monitoring for 12 months. This equates to seven appointments over the first 3 months. We thus decided that participants in our trial would have seven individual dietary appointments over 16 weeks, as participants would be in contact with us on other occasions for measurement of trial outcomes, meaning that despite spreading the seven appointments over a longer period than that recommended by the NHMRC, the frequency of contact with our team, including the Accredited Practicing Dietitian, would actually be greater.

The initial individual dietary appointment was scheduled for approximately 90 min, with subsequent review appointments of 30–60 min. We would allow participants the option of doing some of their individual dietary appointments over the phone, because many of the women in our trial would most likely still be working and may find it difficult to attend the clinic in person for every appointment and because the efficacy of dietary interventions delivered by telephone is supported by evidence 37. Individual dietary appointments would provide a comprehensive weight management consultation that, in addition to the dietary intervention, addresses key aspects of lifestyle modification, namely behaviour change and physical activity. Although the TEMPO Diet Trial is not an intervention that specifically investigates physical activity, participants in both interventions would be given a pedometer and general advice to gradually increase daily step counts to 8000–12,000 steps per day, including 30–60 min per day of moderate to vigorous physical activity. This recommendation was based on achieving 200–300 min per week of physical activity, as recommended in the 2009 American College of Sports Physicians guidelines for weight loss and prevention of weight gain 38.

## Conclusion

The purpose of this paper was to present the underlying rationale and development process behind the dietary interventions to be used in the TEMPO Diet Trial. Whilst the TEMPO Diet Trial is more of an efficacy study than an effectiveness study, as it is conducted in a research setting and in a narrowly defined population, it has been designed to maximize clinical utility by drawing on existing resources and clinical practice guidelines. This work has demonstrated that there are several different steps and considerations that lead to the final intervention – which is rarely reported in the literature but which could be useful to readers of outcome papers or to those wishing to design their own dietary weight loss interventions for research or clinical purposes. Reporting more detailed information about the design and characteristics of dietary interventions could help to bridge the gap in translating findings from research into clinical practice.

## Conflict of Interest Statement

For the TEMPO Diet Trial, Prima Health Solutions, Sydney Australia, provided in‐kind support in the form of below cost KicStart™ VLED and a gift of associated adherence tools (shakers). They had no involvement in the design or analysis of the research. This relationship with Prima Health Solutions was established after the modelling in this paper had been undertaken.

JF has received payment from Eli Lilly, the Pharmacy Guild of Australia, Micare, iNova Pharmaceuticals and Novo Nordisk for seminar presentations to clinicians about obesity. She consulted for Nestlé (Optifast) Australia from 2005 to 2011.

IDC performs clinical trials of obesity treatment and prevention, some of which have been funded by government, but others by the pharmaceutical industry. Current trials are funded by Novo Nordisk, Pfizer, Bristol‐Myers Squibb and Soho Flordis International. He serves on the steering committees of the EXSCEL trial, for which he receives an honorarium from the sponsor, Astra Zeneca. He has given talks for Novo Nordisk, Servier Laboratories, Ache and Pfizer in the last 3 years.

AS has received payment from Eli Lilly, the Pharmacy Guild of Australia, Novo Nordisk and the Dietitians Association of Australia for seminar presentations at conferences. She is also the author of *The Don't Go Hungry Diet* (Bantam, Australia and New Zealand, 2007) and *Don't Go Hungry For Life* (Bantam, Australia and New Zealand, 2011).

TPM has received research grants for clinical trials funded by the Australian Egg Corporation, GlaxoSmithKline, Novo Nordisk, Pfizer, Roche, Weight Watchers and Zafgen. TPM acts as an advisory member to the Egg Nutrition Council, Nestlé Nutrition and Novo Nordisk and has received payments for lectures from Novo Nordisk and Astra Zeneca.

## Funding

This work was supported by the National Health and Medical Research Council (NHMRC) of Australia via an Early Career Research Fellowship to RVS (1072771), a project grant to AS, NMB and IDC (1026005), a program grant to IDC (1037786) and a Senior Research Fellowship to AS (1042555). We are also grateful to the Endocrine Society of Australia for a Postdoctoral Award to RVS and to the Australian Research Council for an Australian Postgraduate Award to AAG.

## Supporting information


**Figure S1**. Height (in m, horizontal axis) and weight (in kg, vertical axis) of potentially eligible (shaded) and ineligible (unshaded) women based on body mass index criteria of 30–40 kg m^−2^ for women between 1.5and 1.7 m tall.
**Figure S2**. Estimated energy expenditure (EEE, in MJ per day) of potentially eligible women (body mass index 30–40 kg m^−2^) according to height (in m, horizontal axis) and weight (in kg, vertical axis) calculated with the Harris–Benedict equation using adjusted ideal body weight and a physical activity level of 1.4. Only EEE of women in the eligible range of height and weight are shown.

Supporting info item
