# Supplementary material for: Fast versus slow weight loss: development process and rationale behind the dietary interventions for the TEMPO Diet Trial
Source: Obes Sci Pract. 2016 May 31;2(2):162–73. doi: 10.1002/osp4.48 (PMC5089659; doi:10.1002/osp4.48)
Supplement: Supplementary file 1 — Supporting info item [file OSP4-2-162-s001.docx]

**Supporting information**

**Fast versus slow weight loss: development process and rationale behind the dietary interventions for the TEMPO Diet Trial**

Alice A Gibson^a^, Radhika V Seimon^a^, Janet Franklin^a^, Tania P Markovic^a,b^, Nuala M Byrne^c^, Elisia Manson^b^, Ian D Caterson^a,b^ & Amanda Sainsbury^a^

^a^Boden Institute of Obesity, Nutrition, Exercise & Eating Disorders, Sydney Medical School, Charles Perkins Centre, University of Sydney, NSW, Australia, 2006

^b^Metabolism & Obesity Services, Royal Prince Alfred Hospital, Camperdown, NSW, Australia, 2050

^c^Bond Institute of Health and Sport, Faculty of Health Sciences and Medicine, Bond University, Gold Coast, Australia, 4226

**Corresponding author:**

Alice Gibson

Boden Institute of Obesity Nutrition Exercise & Eating Disorders, Charles Perkins Centre, University of Sydney, NSW 2006. [alice.gibson@sydney.edu.au](mailto:alice.gibson@sydney.edu.au)

**Modeling of potentially eligible participants**

Nutritional requirements differ for persons of different age, sex, height, weight and physical activity level. Hence, the first consideration in designing the dietary interventions for the TEMPO Diet Trial was to model (or predict) characteristics of the potentially eligible population so that accurate dietary prescriptions and the logistics of implementation could be determined prior to seeing participants.

In 2011 – 2012, the average height of women aged 45 to 64 in Australia was 1.62 m ^1^. Less than 20% were below 1.55 m tall, and less than 10% were above 1.70 m tall ^2^. Therefore, for our model of potentially eligible people we used a lower limit of 1.50 m and an upper limit of 1.70 m for height. This range of height, at 1 cm increments, was then entered into the first row of a Microsoft Excel sheet as shown in **Figure S1**. We then calculated the weight of the lightest potentially eligible person (1.50 m tall and with and body mass index [BMI] of 30 kg/m^2^) as 68 kg, and of the heaviest potentially eligible person (1.70 m tall and with a BMI of 40 kg/m^2^) as 115 kg. Then, in 1 kg increments, we filled down the first column of the Microsoft Excel sheet, also shown in **Figure S1**, starting from 67 kg and continuing until 116 kg.

Using the height in metres (m) along the first row, and weight in kilograms (kg) down the first column, we populated the table using the equation of BMI = weight in kg / height in m^2^. From here we applied conditional formatting so that the eligible weights for each particular height displayed were shaded, and the ineligible weights were not. Thus, the range of weights of potential participants, depending on height, would likely fall between 68 and 115 kg (**Figure S1**). This figure also provides a quick reference tool to determine eligibility, which may be useful, for example when conducting telephone screening.

Having identified the characteristics of our potentially eligible population (age, sex, height, weight and physical activity level), we could then model the predicted energy requirements in order to determine dietary energy prescriptions for the intervention groups. We calculated estimated energy expenditure (EEE) using the Harris Benedict Equation for all potentially eligible women ^3^ (**Figure S2**):

**EEE = REE x PAL**

*Where: REE = resting energy expenditure, PAL = physical activity level*

**REE = 2741 + (40.00 x W) + ((7.74 x H)/100) – (19.56 x A)**

*Where: W = weight in kg, H = height in m and A = age in years*

We used a conservative PAL of 1.4 for all calculations, as this represents the lower end of the sedentary to light physical activity levels outlined by the Food and Agricultural Organization of the United Nations (FAO), the World Health Organisation (WHO) and the United Nations University (UNU) ^4^. We used an age of 55 years for all calculations (midpoint of our 45-65 year eligible age range), as REE has been shown to reduce by only about 1-2% per decade ^5^.

For weight we did not use actual body weight but instead used an *adjusted ideal body weight (AIBW)^6^*:

**AIBW= [(actual body weight – IBW) x 0.25] + IBW**

*Where: IBW = ideal body weight, which is body weight at a BMI of 25 kg/m^2^, and 0.25 is an estimate % of additional metabolically active body weight.*

We used AIBW instead of actual body weight for the calculation of EEE because the Harris Benedict equation has been shown to overestimate the energy requirements of individuals with obesity when actual body weight is used, and to underestimate energy requirements when IBW is used ^7, 8^. The above-mentioned adjustment attempts to account for the disproportionate increase in fat mass in people with obesity, compared with fat free mass which is more metabolically active ^9, 10^.

|  | **1.50** | **1.51** | **1.52** | **1.53** | **1.54** | **1.55** | **1.56** | **1.57** | **1.58** | **1.59** | **1.60** | **1.61** | **1.62** | **1.63** | **1.64** | **1.65** | **1.66** | **1.67** | **1.68** | **1.69** | **1.70** |
| --- | --- | --- | --- | --- | --- | --- | --- | --- | --- | --- | --- | --- | --- | --- | --- | --- | --- | --- | --- | --- | --- |
| **67** | 29.8 | 29.4 | 29.0 | 28.6 | 28.3 | 27.9 | 27.5 | 27.2 | 26.8 | 26.5 | 26.2 | 25.8 | 25.5 | 25.2 | 24.9 | 24.6 | 24.3 | 24.0 | 23.7 | 23.5 | 23.2 |
| **68** | 30.2 | 29.8 | 29.4 | 29.0 | 28.7 | 28.3 | 27.9 | 27.6 | 27.2 | 26.9 | 26.6 | 26.2 | 25.9 | 25.6 | 25.3 | 25.0 | 24.7 | 24.4 | 24.1 | 23.8 | 23.5 |
| **69** | 30.7 | 30.3 | 29.9 | 29.5 | 29.1 | 28.7 | 28.4 | 28.0 | 27.6 | 27.3 | 27.0 | 26.6 | 26.3 | 26.0 | 25.7 | 25.3 | 25.0 | 24.7 | 24.4 | 24.2 | 23.9 |
| **70** | 31.1 | 30.7 | 30.3 | 29.9 | 29.5 | 29.1 | 28.8 | 28.4 | 28.0 | 27.7 | 27.3 | 27.0 | 26.7 | 26.3 | 26.0 | 25.7 | 25.4 | 25.1 | 24.8 | 24.5 | 24.2 |
| **71** | 31.6 | 31.1 | 30.7 | 30.3 | 29.9 | 29.6 | 29.2 | 28.8 | 28.4 | 28.1 | 27.7 | 27.4 | 27.1 | 26.7 | 26.4 | 26.1 | 25.8 | 25.5 | 25.2 | 24.9 | 24.6 |
| **72** | 32.0 | 31.6 | 31.2 | 30.8 | 30.4 | 30.0 | 29.6 | 29.2 | 28.8 | 28.5 | 28.1 | 27.8 | 27.4 | 27.1 | 26.8 | 26.4 | 26.1 | 25.8 | 25.5 | 25.2 | 24.9 |
| **73** | 32.4 | 32.0 | 31.6 | 31.2 | 30.8 | 30.4 | 30.0 | 29.6 | 29.2 | 28.9 | 28.5 | 28.2 | 27.8 | 27.5 | 27.1 | 26.8 | 26.5 | 26.2 | 25.9 | 25.6 | 25.3 |
| **74** | 32.9 | 32.5 | 32.0 | 31.6 | 31.2 | 30.8 | 30.4 | 30.0 | 29.6 | 29.3 | 28.9 | 28.5 | 28.2 | 27.9 | 27.5 | 27.2 | 26.9 | 26.5 | 26.2 | 25.9 | 25.6 |
| **75** | 33.3 | 32.9 | 32.5 | 32.0 | 31.6 | 31.2 | 30.8 | 30.4 | 30.0 | 29.7 | 29.3 | 28.9 | 28.6 | 28.2 | 27.9 | 27.5 | 27.2 | 26.9 | 26.6 | 26.3 | 26.0 |
| **76** | 33.8 | 33.3 | 32.9 | 32.5 | 32.0 | 31.6 | 31.2 | 30.8 | 30.4 | 30.1 | 29.7 | 29.3 | 29.0 | 28.6 | 28.3 | 27.9 | 27.6 | 27.3 | 26.9 | 26.6 | 26.3 |
| **77** | 34.2 | 33.8 | 33.3 | 32.9 | 32.5 | 32.0 | 31.6 | 31.2 | 30.8 | 30.5 | 30.1 | 29.7 | 29.3 | 29.0 | 28.6 | 28.3 | 27.9 | 27.6 | 27.3 | 27.0 | 26.6 |
| **78** | 34.7 | 34.2 | 33.8 | 33.3 | 32.9 | 32.5 | 32.1 | 31.6 | 31.2 | 30.9 | 30.5 | 30.1 | 29.7 | 29.4 | 29.0 | 28.7 | 28.3 | 28.0 | 27.6 | 27.3 | 27.0 |
| **79** | 35.1 | 34.6 | 34.2 | 33.7 | 33.3 | 32.9 | 32.5 | 32.0 | 31.6 | 31.2 | 30.9 | 30.5 | 30.1 | 29.7 | 29.4 | 29.0 | 28.7 | 28.3 | 28.0 | 27.7 | 27.3 |
| **80** | 35.6 | 35.1 | 34.6 | 34.2 | 33.7 | 33.3 | 32.9 | 32.5 | 32.0 | 31.6 | 31.3 | 30.9 | 30.5 | 30.1 | 29.7 | 29.4 | 29.0 | 28.7 | 28.3 | 28.0 | 27.7 |
| **81** | 36.0 | 35.5 | 35.1 | 34.6 | 34.2 | 33.7 | 33.3 | 32.9 | 32.4 | 32.0 | 31.6 | 31.2 | 30.9 | 30.5 | 30.1 | 29.8 | 29.4 | 29.0 | 28.7 | 28.4 | 28.0 |
| **82** | 36.4 | 36.0 | 35.5 | 35.0 | 34.6 | 34.1 | 33.7 | 33.3 | 32.8 | 32.4 | 32.0 | 31.6 | 31.2 | 30.9 | 30.5 | 30.1 | 29.8 | 29.4 | 29.1 | 28.7 | 28.4 |
| **83** | 36.9 | 36.4 | 35.9 | 35.5 | 35.0 | 34.5 | 34.1 | 33.7 | 33.2 | 32.8 | 32.4 | 32.0 | 31.6 | 31.2 | 30.9 | 30.5 | 30.1 | 29.8 | 29.4 | 29.1 | 28.7 |
| **84** | 37.3 | 36.8 | 36.4 | 35.9 | 35.4 | 35.0 | 34.5 | 34.1 | 33.6 | 33.2 | 32.8 | 32.4 | 32.0 | 31.6 | 31.2 | 30.9 | 30.5 | 30.1 | 29.8 | 29.4 | 29.1 |
| **85** | 37.8 | 37.3 | 36.8 | 36.3 | 35.8 | 35.4 | 34.9 | 34.5 | 34.0 | 33.6 | 33.2 | 32.8 | 32.4 | 32.0 | 31.6 | 31.2 | 30.8 | 30.5 | 30.1 | 29.8 | 29.4 |
| **86** | 38.2 | 37.7 | 37.2 | 36.7 | 36.3 | 35.8 | 35.3 | 34.9 | 34.4 | 34.0 | 33.6 | 33.2 | 32.8 | 32.4 | 32.0 | 31.6 | 31.2 | 30.8 | 30.5 | 30.1 | 29.8 |
| **87** | 38.7 | 38.2 | 37.7 | 37.2 | 36.7 | 36.2 | 35.7 | 35.3 | 34.9 | 34.4 | 34.0 | 33.6 | 33.2 | 32.7 | 32.3 | 32.0 | 31.6 | 31.2 | 30.8 | 30.5 | 30.1 |
| **88** | 39.1 | 38.6 | 38.1 | 37.6 | 37.1 | 36.6 | 36.2 | 35.7 | 35.3 | 34.8 | 34.4 | 33.9 | 33.5 | 33.1 | 32.7 | 32.3 | 31.9 | 31.6 | 31.2 | 30.8 | 30.4 |
| **89** | 39.6 | 39.0 | 38.5 | 38.0 | 37.5 | 37.0 | 36.6 | 36.1 | 35.7 | 35.2 | 34.8 | 34.3 | 33.9 | 33.5 | 33.1 | 32.7 | 32.3 | 31.9 | 31.5 | 31.2 | 30.8 |
| **90** | 40.0 | 39.5 | 39.0 | 38.4 | 37.9 | 37.5 | 37.0 | 36.5 | 36.1 | 35.6 | 35.2 | 34.7 | 34.3 | 33.9 | 33.5 | 33.1 | 32.7 | 32.3 | 31.9 | 31.5 | 31.1 |
| **91** | 40.4 | 39.9 | 39.4 | 38.9 | 38.4 | 37.9 | 37.4 | 36.9 | 36.5 | 36.0 | 35.5 | 35.1 | 34.7 | 34.3 | 33.8 | 33.4 | 33.0 | 32.6 | 32.2 | 31.9 | 31.5 |
| **92** | 40.9 | 40.3 | 39.8 | 39.3 | 38.8 | 38.3 | 37.8 | 37.3 | 36.9 | 36.4 | 35.9 | 35.5 | 35.1 | 34.6 | 34.2 | 33.8 | 33.4 | 33.0 | 32.6 | 32.2 | 31.8 |
| **93** | 41.3 | 40.8 | 40.3 | 39.7 | 39.2 | 38.7 | 38.2 | 37.7 | 37.3 | 36.8 | 36.3 | 35.9 | 35.4 | 35.0 | 34.6 | 34.2 | 33.7 | 33.3 | 33.0 | 32.6 | 32.2 |
| **94** | 41.8 | 41.2 | 40.7 | 40.2 | 39.6 | 39.1 | 38.6 | 38.1 | 37.7 | 37.2 | 36.7 | 36.3 | 35.8 | 35.4 | 34.9 | 34.5 | 34.1 | 33.7 | 33.3 | 32.9 | 32.5 |
| **95** | 42.2 | 41.7 | 41.1 | 40.6 | 40.1 | 39.5 | 39.0 | 38.5 | 38.1 | 37.6 | 37.1 | 36.6 | 36.2 | 35.8 | 35.3 | 34.9 | 34.5 | 34.1 | 33.7 | 33.3 | 32.9 |
| **96** | 42.7 | 42.1 | 41.6 | 41.0 | 40.5 | 40.0 | 39.4 | 38.9 | 38.5 | 38.0 | 37.5 | 37.0 | 36.6 | 36.1 | 35.7 | 35.3 | 34.8 | 34.4 | 34.0 | 33.6 | 33.2 |
| **97** | 43.1 | 42.5 | 42.0 | 41.4 | 40.9 | 40.4 | 39.9 | 39.4 | 38.9 | 38.4 | 37.9 | 37.4 | 37.0 | 36.5 | 36.1 | 35.6 | 35.2 | 34.8 | 34.4 | 34.0 | 33.6 |
| **98** | 43.6 | 43.0 | 42.4 | 41.9 | 41.3 | 40.8 | 40.3 | 39.8 | 39.3 | 38.8 | 38.3 | 37.8 | 37.3 | 36.9 | 36.4 | 36.0 | 35.6 | 35.1 | 34.7 | 34.3 | 33.9 |
| **99** | 44.0 | 43.4 | 42.8 | 42.3 | 41.7 | 41.2 | 40.7 | 40.2 | 39.7 | 39.2 | 38.7 | 38.2 | 37.7 | 37.3 | 36.8 | 36.4 | 35.9 | 35.5 | 35.1 | 34.7 | 34.3 |
| **100** | 44.4 | 43.9 | 43.3 | 42.7 | 42.2 | 41.6 | 41.1 | 40.6 | 40.1 | 39.6 | 39.1 | 38.6 | 38.1 | 37.6 | 37.2 | 36.7 | 36.3 | 35.9 | 35.4 | 35.0 | 34.6 |
| **101** | 44.9 | 44.3 | 43.7 | 43.1 | 42.6 | 42.0 | 41.5 | 41.0 | 40.5 | 40.0 | 39.5 | 39.0 | 38.5 | 38.0 | 37.6 | 37.1 | 36.7 | 36.2 | 35.8 | 35.4 | 34.9 |
| **102** | 45.3 | 44.7 | 44.1 | 43.6 | 43.0 | 42.5 | 41.9 | 41.4 | 40.9 | 40.3 | 39.8 | 39.4 | 38.9 | 38.4 | 37.9 | 37.5 | 37.0 | 36.6 | 36.1 | 35.7 | 35.3 |
| **103** | 45.8 | 45.2 | 44.6 | 44.0 | 43.4 | 42.9 | 42.3 | 41.8 | 41.3 | 40.7 | 40.2 | 39.7 | 39.2 | 38.8 | 38.3 | 37.8 | 37.4 | 36.9 | 36.5 | 36.1 | 35.6 |
| **104** | 46.2 | 45.6 | 45.0 | 44.4 | 43.9 | 43.3 | 42.7 | 42.2 | 41.7 | 41.1 | 40.6 | 40.1 | 39.6 | 39.1 | 38.7 | 38.2 | 37.7 | 37.3 | 36.8 | 36.4 | 36.0 |
| **105** | 46.7 | 46.1 | 45.4 | 44.9 | 44.3 | 43.7 | 43.1 | 42.6 | 42.1 | 41.5 | 41.0 | 40.5 | 40.0 | 39.5 | 39.0 | 38.6 | 38.1 | 37.6 | 37.2 | 36.8 | 36.3 |
| **106** | 47.1 | 46.5 | 45.9 | 45.3 | 44.7 | 44.1 | 43.6 | 43.0 | 42.5 | 41.9 | 41.4 | 40.9 | 40.4 | 39.9 | 39.4 | 38.9 | 38.5 | 38.0 | 37.6 | 37.1 | 36.7 |
| **107** | 47.6 | 46.9 | 46.3 | 45.7 | 45.1 | 44.5 | 44.0 | 43.4 | 42.9 | 42.3 | 41.8 | 41.3 | 40.8 | 40.3 | 39.8 | 39.3 | 38.8 | 38.4 | 37.9 | 37.5 | 37.0 |
| **108** | 48.0 | 47.4 | 46.7 | 46.1 | 45.5 | 45.0 | 44.4 | 43.8 | 43.3 | 42.7 | 42.2 | 41.7 | 41.2 | 40.6 | 40.2 | 39.7 | 39.2 | 38.7 | 38.3 | 37.8 | 37.4 |
| **109** | 48.4 | 47.8 | 47.2 | 46.6 | 46.0 | 45.4 | 44.8 | 44.2 | 43.7 | 43.1 | 42.6 | 42.1 | 41.5 | 41.0 | 40.5 | 40.0 | 39.6 | 39.1 | 38.6 | 38.2 | 37.7 |
| **110** | 48.9 | 48.2 | 47.6 | 47.0 | 46.4 | 45.8 | 45.2 | 44.6 | 44.1 | 43.5 | 43.0 | 42.4 | 41.9 | 41.4 | 40.9 | 40.4 | 39.9 | 39.4 | 39.0 | 38.5 | 38.1 |
| **111** | 49.3 | 48.7 | 48.0 | 47.4 | 46.8 | 46.2 | 45.6 | 45.0 | 44.5 | 43.9 | 43.4 | 42.8 | 42.3 | 41.8 | 41.3 | 40.8 | 40.3 | 39.8 | 39.3 | 38.9 | 38.4 |
| **112** | 49.8 | 49.1 | 48.5 | 47.8 | 47.2 | 46.6 | 46.0 | 45.4 | 44.9 | 44.3 | 43.8 | 43.2 | 42.7 | 42.2 | 41.6 | 41.1 | 40.6 | 40.2 | 39.7 | 39.2 | 38.8 |
| **113** | 50.2 | 49.6 | 48.9 | 48.3 | 47.6 | 47.0 | 46.4 | 45.8 | 45.3 | 44.7 | 44.1 | 43.6 | 43.1 | 42.5 | 42.0 | 41.5 | 41.0 | 40.5 | 40.0 | 39.6 | 39.1 |
| **114** | 50.7 | 50.0 | 49.3 | 48.7 | 48.1 | 47.5 | 46.8 | 46.2 | 45.7 | 45.1 | 44.5 | 44.0 | 43.4 | 42.9 | 42.4 | 41.9 | 41.4 | 40.9 | 40.4 | 39.9 | 39.4 |
| **115** | 51.1 | 50.4 | 49.8 | 49.1 | 48.5 | 47.9 | 47.3 | 46.7 | 46.1 | 45.5 | 44.9 | 44.4 | 43.8 | 43.3 | 42.8 | 42.2 | 41.7 | 41.2 | 40.7 | 40.3 | 39.8 |
| **116** | 51.6 | 50.9 | 50.2 | 49.6 | 48.9 | 48.3 | 47.7 | 47.1 | 46.5 | 45.9 | 45.3 | 44.8 | 44.2 | 43.7 | 43.1 | 42.6 | 42.1 | 41.6 | 41.1 | 40.6 | 40.1 |

**Figure S1.** Height (in m, horizontal axis) and weight (in kg, vertical axis) of potentially eligible (shaded) and ineligible (unshaded) women based on body mass index criteria of 30-40 kg/m^2^ for women between 1.5 m and 1.7 m tall.

|  | **1.50** | **1.51** | **1.52** | **1.53** | **1.54** | **1.55** | **1.56** | **1.57** | **1.58** | **1.59** | **1.60** | **1.61** | **1.62** | **1.63** | **1.64** | **1.65** | **1.66** | **1.67** | **1.68** | **1.69** | **1.70** |
| --- | --- | --- | --- | --- | --- | --- | --- | --- | --- | --- | --- | --- | --- | --- | --- | --- | --- | --- | --- | --- | --- |
| **67** |  |  |  |  |  |  |  |  |  |  |  |  |  |  |  |  |  |  |  |  |  |
| **68** | 9.1 |  |  |  |  |  |  |  |  |  |  |  |  |  |  |  |  |  |  |  |  |
| **69** | 9.1 | 9.1 |  |  |  |  |  |  |  |  |  |  |  |  |  |  |  |  |  |  |  |
| **70** | 9.1 | 9.1 | 9.2 |  |  |  |  |  |  |  |  |  |  |  |  |  |  |  |  |  |  |
| **71** | 9.1 | 9.1 | 9.2 | 9.2 |  |  |  |  |  |  |  |  |  |  |  |  |  |  |  |  |  |
| **72** | 9.1 | 9.2 | 9.2 | 9.2 | 9.3 |  |  |  |  |  |  |  |  |  |  |  |  |  |  |  |  |
| **73** | 9.1 | 9.2 | 9.2 | 9.3 | 9.3 | 9.3 |  |  |  |  |  |  |  |  |  |  |  |  |  |  |  |
| **74** | 9.1 | 9.2 | 9.2 | 9.3 | 9.3 | 9.4 | 9.4 | 9.4 |  |  |  |  |  |  |  |  |  |  |  |  |  |
| **75** | 9.2 | 9.2 | 9.2 | 9.3 | 9.3 | 9.4 | 9.4 | 9.5 | 9.5 |  |  |  |  |  |  |  |  |  |  |  |  |
| **76** | 9.2 | 9.2 | 9.3 | 9.3 | 9.3 | 9.4 | 9.4 | 9.5 | 9.5 | 9.6 |  |  |  |  |  |  |  |  |  |  |  |
| **77** | 9.2 | 9.2 | 9.3 | 9.3 | 9.4 | 9.4 | 9.4 | 9.5 | 9.5 | 9.6 | 9.6 |  |  |  |  |  |  |  |  |  |  |
| **78** | 9.2 | 9.2 | 9.3 | 9.3 | 9.4 | 9.4 | 9.5 | 9.5 | 9.5 | 9.6 | 9.6 | 9.7 |  |  |  |  |  |  |  |  |  |
| **79** | 9.2 | 9.3 | 9.3 | 9.3 | 9.4 | 9.4 | 9.5 | 9.5 | 9.6 | 9.6 | 9.7 | 9.7 | 9.7 |  |  |  |  |  |  |  |  |
| **80** | 9.2 | 9.3 | 9.3 | 9.4 | 9.4 | 9.4 | 9.5 | 9.5 | 9.6 | 9.6 | 9.7 | 9.7 | 9.8 | 9.8 |  |  |  |  |  |  |  |
| **81** | 9.2 | 9.3 | 9.3 | 9.4 | 9.4 | 9.5 | 9.5 | 9.5 | 9.6 | 9.6 | 9.7 | 9.7 | 9.8 | 9.8 | 9.9 |  |  |  |  |  |  |
| **82** | 9.3 | 9.3 | 9.3 | 9.4 | 9.4 | 9.5 | 9.5 | 9.6 | 9.6 | 9.6 | 9.7 | 9.7 | 9.8 | 9.8 | 9.9 | 9.9 |  |  |  |  |  |
| **83** | 9.3 | 9.3 | 9.4 | 9.4 | 9.4 | 9.5 | 9.5 | 9.6 | 9.6 | 9.7 | 9.7 | 9.8 | 9.8 | 9.8 | 9.9 | 9.9 | 10.0 |  |  |  |  |
| **84** | 9.3 | 9.3 | 9.4 | 9.4 | 9.5 | 9.5 | 9.5 | 9.6 | 9.6 | 9.7 | 9.7 | 9.8 | 9.8 | 9.9 | 9.9 | 9.9 | 10.0 | 10.0 |  |  |  |
| **85** | 9.3 | 9.3 | 9.4 | 9.4 | 9.5 | 9.5 | 9.6 | 9.6 | 9.6 | 9.7 | 9.7 | 9.8 | 9.8 | 9.9 | 9.9 | 10.0 | 10.0 | 10.1 | 10.1 |  |  |
| **86** | 9.3 | 9.4 | 9.4 | 9.4 | 9.5 | 9.5 | 9.6 | 9.6 | 9.7 | 9.7 | 9.7 | 9.8 | 9.8 | 9.9 | 9.9 | 10.0 | 10.0 | 10.1 | 10.1 | 10.2 |  |
| **87** | 9.3 | 9.4 | 9.4 | 9.5 | 9.5 | 9.5 | 9.6 | 9.6 | 9.7 | 9.7 | 9.8 | 9.8 | 9.9 | 9.9 | 9.9 | 10.0 | 10.0 | 10.1 | 10.1 | 10.2 | 10.2 |
| **88** | 9.3 | 9.4 | 9.4 | 9.5 | 9.5 | 9.6 | 9.6 | 9.6 | 9.7 | 9.7 | 9.8 | 9.8 | 9.9 | 9.9 | 10.0 | 10.0 | 10.0 | 10.1 | 10.1 | 10.2 | 10.2 |
| **89** | 9.4 | 9.4 | 9.4 | 9.5 | 9.5 | 9.6 | 9.6 | 9.7 | 9.7 | 9.7 | 9.8 | 9.8 | 9.9 | 9.9 | 10.0 | 10.0 | 10.1 | 10.1 | 10.2 | 10.2 | 10.2 |
| **90** | 9.4 | 9.4 | 9.5 | 9.5 | 9.5 | 9.6 | 9.6 | 9.7 | 9.7 | 9.8 | 9.8 | 9.8 | 9.9 | 9.9 | 10.0 | 10.0 | 10.1 | 10.1 | 10.2 | 10.2 | 10.3 |
| **91** |  | 9.4 | 9.5 | 9.5 | 9.6 | 9.6 | 9.6 | 9.7 | 9.7 | 9.8 | 9.8 | 9.9 | 9.9 | 10.0 | 10.0 | 10.0 | 10.1 | 10.1 | 10.2 | 10.2 | 10.3 |
| **92** |  |  | 9.5 | 9.5 | 9.6 | 9.6 | 9.7 | 9.7 | 9.7 | 9.8 | 9.8 | 9.9 | 9.9 | 10.0 | 10.0 | 10.1 | 10.1 | 10.1 | 10.2 | 10.2 | 10.3 |
| **93** |  |  |  | 9.5 | 9.6 | 9.6 | 9.7 | 9.7 | 9.8 | 9.8 | 9.8 | 9.9 | 9.9 | 10.0 | 10.0 | 10.1 | 10.1 | 10.2 | 10.2 | 10.3 | 10.3 |
| **94** |  |  |  |  | 9.6 | 9.6 | 9.7 | 9.7 | 9.8 | 9.8 | 9.9 | 9.9 | 9.9 | 10.0 | 10.0 | 10.1 | 10.1 | 10.2 | 10.2 | 10.3 | 10.3 |
| **95** |  |  |  |  |  | 9.7 | 9.7 | 9.7 | 9.8 | 9.8 | 9.9 | 9.9 | 10.0 | 10.0 | 10.1 | 10.1 | 10.1 | 10.2 | 10.2 | 10.3 | 10.3 |
| **96** |  |  |  |  |  | 9.7 | 9.7 | 9.8 | 9.8 | 9.8 | 9.9 | 9.9 | 10.0 | 10.0 | 10.1 | 10.1 | 10.2 | 10.2 | 10.3 | 10.3 | 10.3 |
| **97** |  |  |  |  |  |  | 9.7 | 9.8 | 9.8 | 9.9 | 9.9 | 9.9 | 10.0 | 10.0 | 10.1 | 10.1 | 10.2 | 10.2 | 10.3 | 10.3 | 10.4 |
| **98** |  |  |  |  |  |  |  | 9.8 | 9.8 | 9.9 | 9.9 | 10.0 | 10.0 | 10.1 | 10.1 | 10.1 | 10.2 | 10.2 | 10.3 | 10.3 | 10.4 |
| **99** |  |  |  |  |  |  |  |  | 9.8 | 9.9 | 9.9 | 10.0 | 10.0 | 10.1 | 10.1 | 10.2 | 10.2 | 10.2 | 10.3 | 10.3 | 10.4 |
| **100** |  |  |  |  |  |  |  |  |  | 9.9 | 9.9 | 10.0 | 10.0 | 10.1 | 10.1 | 10.2 | 10.2 | 10.3 | 10.3 | 10.4 | 10.4 |
| **101** |  |  |  |  |  |  |  |  |  | 9.9 | 10.0 | 10.0 | 10.0 | 10.1 | 10.1 | 10.2 | 10.2 | 10.3 | 10.3 | 10.4 | 10.4 |
| **102** |  |  |  |  |  |  |  |  |  |  | 10.0 | 10.0 | 10.1 | 10.1 | 10.2 | 10.2 | 10.2 | 10.3 | 10.3 | 10.4 | 10.4 |
| **103** |  |  |  |  |  |  |  |  |  |  |  | 10.0 | 10.1 | 10.1 | 10.2 | 10.2 | 10.3 | 10.3 | 10.3 | 10.4 | 10.4 |
| **104** |  |  |  |  |  |  |  |  |  |  |  |  | 10.1 | 10.1 | 10.2 | 10.2 | 10.3 | 10.3 | 10.4 | 10.4 | 10.5 |
| **105** |  |  |  |  |  |  |  |  |  |  |  |  |  | 10.1 | 10.2 | 10.2 | 10.3 | 10.3 | 10.4 | 10.4 | 10.5 |
| **106** |  |  |  |  |  |  |  |  |  |  |  |  |  | 10.2 | 10.2 | 10.3 | 10.3 | 10.3 | 10.4 | 10.4 | 10.5 |
| **107** |  |  |  |  |  |  |  |  |  |  |  |  |  |  | 10.2 | 10.3 | 10.3 | 10.4 | 10.4 | 10.5 | 10.5 |
| **108** |  |  |  |  |  |  |  |  |  |  |  |  |  |  |  | 10.3 | 10.3 | 10.4 | 10.4 | 10.5 | 10.5 |
| **109** |  |  |  |  |  |  |  |  |  |  |  |  |  |  |  |  | 10.3 | 10.4 | 10.4 | 10.5 | 10.5 |
| **110** |  |  |  |  |  |  |  |  |  |  |  |  |  |  |  |  | 10.4 | 10.4 | 10.4 | 10.5 | 10.5 |
| **111** |  |  |  |  |  |  |  |  |  |  |  |  |  |  |  |  |  | 10.4 | 10.5 | 10.5 | 10.6 |
| **112** |  |  |  |  |  |  |  |  |  |  |  |  |  |  |  |  |  |  | 10.5 | 10.5 | 10.6 |
| **113** |  |  |  |  |  |  |  |  |  |  |  |  |  |  |  |  |  |  |  | 10.5 | 10.6 |
| **114** |  |  |  |  |  |  |  |  |  |  |  |  |  |  |  |  |  |  |  | 10.5 | 10.6 |
| **115** |  |  |  |  |  |  |  |  |  |  |  |  |  |  |  |  |  |  |  |  | 10.6 |
| **116** |  |  |  |  |  |  |  |  |  |  |  |  |  |  |  |  |  |  |  |  |  |

**Figure S2**. Estimated energy expenditure (EEE, in MJ per day) of potentially eligible women (body mass index 30-40 kg/m^2^) according to height (in m, horizontal axis) and weight (in kg, vertical axis) calculated with the Harris Benedict Equation using adjusted ideal body weight and a physical activity level of 1.4. Only EEE of women in the eligible range of height and weight are shown.

**References:**

1 Australian Bureau of Statistics. (2012). 4338.0 - Profiles of Health, Australia, 2011-13: Height and Weight. [WWW document]. Retrieved from <http://www.abs.gov.au/ausstats/abs@.nsf/Lookup/4338.0main+features212011-13>

2 Australian Bureau of Statistics. (2012). 4364.0.55.001 - Australian Health Survey: First Results, 2011-12; Table 14: Height by age and sex- Australia. [WWW document]. Retrieved from <http://www.abs.gov.au/AUSSTATS/abs@.nsf/DetailsPage/4364.0.55.0012011-12?OpenDocument>

3 Harris JA, Benedict FG. A Biometric Study of Human Basal Metabolism. *Proceedings of the National Academy of Sciences of the United States of America*. 1918; 4: 370-73.

4 FAO/WHO/UNU. Human Energy Requirements. A report of a Joint FAO/WHO/UNU Expert consultations. *FAO Food and Nutrition Technical Report Series No 1*. Food and Agriculture Organization: Rome 2004.

5 Keys A, Taylor HL, Grande F. Basal metabolism and age of adult man. *Metabolism: clinical and experimental*. 1973; 22: 579-87.

6 Breen HB, Ireton-Jones CS. Predicting energy needs in obese patients. *Nutr Clin Pract*. 2004; 19: 284-9.

7 Daly JM, Heymsfield SB, Head CA*, et al.* Human energy requirements: overestimation by widely used prediction equation. *The American Journal of Clinical Nutrition*. 1985; 42: 1170-4.

8 Pavlou KN, Hoefer MA, Blackburn GL. Resting energy expenditure in moderate obesity. Predicting velocity of weight loss. *Annals of surgery*. 1986; 203: 136-41.

9 Alves VG, da Rocha EE, Gonzalez MC, da Fonseca RB, Silva MH, Chiesa CA. Assessement of resting energy expenditure of obese patients: comparison of indirect calorimetry with formulae. *Clin Nutr*. 2009; 28: 299-304.

10 da Rocha EE, Alves VG, Silva MH, Chiesa CA, da Fonseca RB. Can measured resting energy expenditure be estimated by formulae in daily clinical nutrition practice? *Curr Opin Clin Nutr Metab Care*. 2005; 8: 319-28.

11 Wadden TA. Treatment of obesity by moderate and severe caloric restriction. Results of clinical research trials. *Ann Intern Med*. 1993; 119: 688-93.
